# Care farming with older people in Europe: policy insights for public health and agriculture on providing care services on farms

**DOI:** 10.3389/phrs.2026.1609528

**Published:** 2026-10-21

**Authors:** Iren Bischofberger, Susann Goerlinger, Mollie Chapman

**Affiliations:** 1 Department of Environmental Systems Sciences, Transdisciplinarity Lab (TdLab), ETH Zürich, Zurich, Switzerland; 2 iilo GmbH, Zürich, Switzerland

**Keywords:** agriculture, care farming, Europe, older adults, transformation

## Abstract

**Background:**

Care farming in Europe, traditionally provided to children and youth with intellectual disabilities, expands to serve older adults due to demographic shifts, providing an alternative to nursing homes and helping farms diversify for sustained businesses. There is a lack of policies which systematically interweave the public health and agricultural sectors. The article provides policy insights for informed decision-making in this transformation.

**Analysis:**

Care farming with older adults involves activities like feeding animals or preparing meals showing positive effects on physical, emotional, and cognitive health, including with dementia, and contrasts sharply with the history of abuse tied to involuntary farm placements.

**Policy Options:**

For intersectoral complexity, a transdisciplinary approach allows to integrate diverse stakeholders across health and agriculture. Policymakers facilitate dialogue to align objectives like production goals versus care farming services.

**Conclusion:**

Sustainable policies must acknowledge complex intersectoral influences by systematically collaborating between health and agriculture sectors through a transdisciplinary approach. Robust practices and models are vital for heterogeneous populations aged 65 and older. A mindset of experimentation and integration among stakeholders supports implementing care farming in a graying society.

## Background

Care farming as a service has been provided in Europe for approximately half a century, primarily to children and youth with disabilities and to people with mental health conditions who live temporarily or permanently on a farm and participate in agricultural activities suited to their capabilities [1]. As European societies experience a demographic shift, care farming for older adults has become a complementary service [2, 3]. Older adults are socio-economically highly heterogeneous (ranging from high to low income, starting from 65 years as approximate pension age to 90 and above), family structures (fewer children living farther away) and the division of reproductive labor (more women in the labor force) lead to different ways of living beyond retirement. These trends go hand in hand with an extended retirement phase in good health, but later also with more years with care needs (e.g., with dementia or Parkinson’s disease). Therefore, facing these typical changing trends for high-income countries, new approaches for living and caregiving in an ageing society are needed. This is where care farming with older persons comes in: older adults who are not part of the farming family spend time temporarily or as permanent residents, while participating in agricultural production or household activities, depending on health conditions and preferences for social interaction. This expansion into a new population group on the farm is accompanied by structural changes affecting both the agricultural and long-term care sectors. The latter is challenged to provide services beyond traditional nursing homes, as baby boomers are particularly eager to live in highly self-determined settings. Municipalities, which are primarily responsible for providing long-term care services, are seeking less costly alternatives to 24/7 nursing homes [4, 5]. In agriculture, small and medium-sized farms face the need to diversify their products and services to sustain their business [6]. Thus, care farming is one solution within multifunctional agriculture to position a farm sustainably in a changing market and demography.

Yet despite these transformations and pockets of innovation around care farming for older adults, the policy infrastructure needed to interweave care and farming is missing. Therefore, in this article, we focus on care farming at the intersection of public health and agriculture. At first glance, this intersection seems to be a good match. However, integrating care and farming requires careful analysis, particularly given that public policies are rarely designed to align the two sectors systematically. The main aim of this article is to provide policy insights from diverse stakeholders across public health and agriculture, so that policymakers and other actors from both sectors can make informed decisions in the current transformation. A specific aim is to lay the groundwork for a roadmap in Switzerland that encompasses both small and large projects to develop care farming sustainably over the next decade. In Switzerland, care farming for older adults is still a niche, with few farms entering the field as early adopters [7] and research only just beginning [8]. Given this early stage of development, we conducted a pilot project alongside this policy paper. It is now timely to start the discourse on care farming for older adults, as nearly 1.6 million of the 9 million population are aged 65 years or older. According to the United Nations Population Division (2023), Switzerland ranks high in Europe’s life expectancy at birth (84 years, 4th after the microstates Monaco, Andorra, and San Marino) [9].

## Analysis

In this chapter, we will first describe how care farming services have evolved in Europe at the intersection of the agricultural and long-term care sectors, followed by an analysis of the underlying normative premises that affect older adults in Western societies and are thus relevant to consider when planning for care farming with older persons.

Farms can begin offering care farming for older adults from different starting points. According to the long experience in the Netherlands [10], the most common scenario is that a farm begins with activities for children and young people who temporarily reside on the farm due to an intellectual disability or during periods of adolescent struggle. Others begin with farm owners’ experiences with kin at age 65 and beyond on the farm. The strategic change to provide services for older persons outside the farming family is often initiated by the farmer’s wife, who is a nurse or a social worker, and who is interested in practicing the profession on the farm and wants to establish an independent income. In one model, farms provide services only during the day, when older adults participate in farming and social activities. Target groups might include older adults who seek to prevent loneliness and employed family members who bring their kin (e.g., parents or grandparents) to the farm [11]. For this day-only service, there is a need for moderate investment, primarily to ensure easy access to and on the farm. Other farms may choose to focus more strongly on care services, collaborate with a long-term care facility, and rent out permanent spaces on the farm grounds. This decision requires a substantial investment in infrastructure for older adults in need of 24/7 care. Again, on other farms, buildings may be empty due to changes in agricultural production. Thus, care farming could be an option for redesigning buildings and services and shifting agricultural production towards more care services [7]. A policy-relevant issue in this case is whether care farming and the production resulting from this service qualify as agricultural activities and thus permit the use of certain buildings. The rental of space or the booking of activities on the farm generates income from care farming with older people.

Typical activities across the care farming types mentioned above might include participation in feeding animals, caring for plants, processing vegetables, and helping prepare meals for the farming team, under the guidance of farm owners or staff [12]. Additionally, participating in health-prevention activities, such as cooking classes on healthy eating or fall-prevention courses, can be part of care farming. Social care on the farm entails spending time with the farming community, such as sharing meals or playing games. In contrast to financing care farming for children and youth, where care farming is usually reimbursed by social insurance, care farming for older adults is often paid out-of-pocket (except for home care services, which are usually covered by health insurance). In countries with a long history of care farming, such as the Netherlands, social security options might be in place to cover care farming activities with older persons [13]. However, in other European countries, coverage through long-term care insurance has not been evaluated so far for care farming with older adults. Yet, a program on “Social farming as a tool for innovation in agriculture” is currently being discussed at the European Union Economic and Social Committee, covering also care farming with older people [14].

Forms of care farming with older people have evolved over time, shaped by local circumstances of multifunctional agriculture and care regimes. Research projects on care farming for older persons–mainly from the Netherlands and Norway–indicate positive effects on older adults’ physical, emotional, cognitive, and social state of health [15, 16]. Particularly, people with dementia has been studied extensively, indicating that care farming fosters the quality of life of patients and family caregivers [17–20]. A review of grey literature reveals case studies from European countries that document care farming initiatives, integrating their specific histories and developments. In Portugal, for instance, emigrants from the 1980s are returning several decades later, after retirement, to their home country and adopting innovative farming practices with older persons in the rural villages where they had lived before emigration. Another example comes from Ireland, where a retired couple remains on the farm after their children have left home. The couple sought an older person who wanted to spend time on the farm during the day, away from the assisted living facility (www.farmelder.eu; additional country reports from Slovenia and Germany). An example of a care farm in Switzerland providing services for older adults is described in Box 1.

BOX 1 **Example of an operational model of care farming with older people (Hadorn & Sokac, 2024, a real example from Switzerland)**

A glimpse on an intergenerational care farm.

In 2018, the daughter of farmers inherited a farm that held mainly cows and pigs. Due to falling milk prices, high operating costs, and limited scope for economic optimization, she transformed the pig barn into a day care center which aims to foster intergenerational encounters of older people and children. In 2023, she founded an association that runs the care farming activities. After taking out a mortgage with the bank, the barn is now wheelchair accessible, contains a living room with a kitchen, a toilet, and a relaxing room. Older persons can spend entire days on the farm (3 days are offered per week), and children (2.5 years and above) and stay three mornings of the same days (option with lunch). There are various activities in the cow barn, garden, or outdoors such as cooking together, processing milk, vegetables, and fruits, as well as enjoying each other’s company. For these activities, professional staff with health and social care background collaborate with volunteers. Transportation to the farm is offered. Payment is out-of-pocket.

These examples from various European countries provide insights into different ways of living on a farm as an older adult, embedded in local history and culture, as well as in natural surroundings that vary in topography and climate. Research literature and country case studies highlight similar epidemiological, demographic, and social challenges: small-scale, nature-based environments on farms provide space for care and health promotion activities for older people within social structures and the farm’s specific agricultural production. Here, older adults can access alternatives to standard care provided by home care services or nursing homes and assisted living facilities.

Care farming, as described so far, must be understood based on underlying normative premises that are deeply embedded in contemporary Western societies about the population of older people (Busch, 2020):

- Firstly, active ageing as a normative request: Old age is often viewed negatively and associated with high healthcare costs, shouldered by society. Therefore, “best agers” are implicitly expected to engage in health promotion and maintain social contacts to reduce loneliness and to prevent increases in healthcare costs and service use.
- Secondly, the family is the responsible authority for care tasks: Despite changing family structures, smaller families, and family members living farther apart, kin are still the first point of contact for older persons living in their own home. Close friends and neighbors might step in to provide support, but it is unclear how sustainable these networks are over more extended periods of need.
- Thirdly, rurality as a synonym for a bygone idyll: The view of intact family structures and traditional ways of living is particularly present in rural regions. Hence, ageing in place in a natural environment is a predominant perspective on retirement, even though most people in Western societies live in urban, particularly suburban, areas. Also, it should be noted at this point, that drawing this idyllic and healing picture of care farms is in contrast to an era in history in the 19th century up to the 1970ies, when children, adults or older persons from marginalized population groups (e.g., children from single mothers) were placed on farms without consent and mistreated as cheap manpower and “morally fallen” subjects [21].

Reflecting on these premises offers an opportunity to rethink and redesign gerontological care and agricultural production during periods of transformation.

## Policy options

Facilitating the development of care farming with older people and ensuring that this occurs in a way that preserves benefits and provides guardrails against risks will require new policies in most countries. In this section, we describe the various actors to consider when developing and establishing care farming for older adults, as well as the key considerations for policymakers. We use Switzerland as our case example (where our expertise and project are based), but we anticipate that insights will foster the discourse in the European context. We are aware of a limiting factor in this regard, since in low- and middle-income countries, active aging is not a normative demand but a survival necessity due to lack of pension schemes. In such social and political contexts, the meaning of care farming changes if older persons have no choice but to work, including on farms. Hence, families are the main support network for older people, or young persons return from further away or even from abroad to take care of grandparents. Also, care farming could blur into forced labor or subsistence farming disguised as therapy. Therefore, good practice of care farming with older adults as described in this article requires basic regulatory frameworks, minimum social protection, and capacity for multi-stakeholder coordination.

Given the intersectoral nature of care farming with older persons, we suggest that a transdisciplinary (td) approach is the most effective way to integrate the diverse needs and expertise of this growing area. Transdisciplinarity serves as a scientific approach to study pressing societal problems, particularly in eras of transformation [22]. The core aim is to bridge the gap between the scientific body of knowledge and the search of social groups for solutions to contemporary challenges. Transdisciplinary methods involve systematic problem framing, the integration of perspectives, and collaboration with various actors in the field through multistakeholder dialogues [23].

In line with this td-approach, a list of potential stakeholders (see Table 1) on a wide range of local and federal levels, from administration to organizations and professional bodies, is typically addressed in the context of care farming with older adults:

**TABLE 1:** List of stakeholders typically involved in care farming with older people (Switzerland, 2026).

Sector	Actor	Example of Switzerland
Public Health/Long-term care	Care facility	Nursing home, home care service, assisted living facility
	Association	Association of care services for older persons
	Administration	Departments of health and social security (municipal, state, and federal level), including for social pension schemes or cash-for-care programs
Agriculture	Farm	Farm providing care services for older adults
	Care farming organization	Organization providing support for farms starting and evolving to offer care services
	Association	Farmers’ association (national, local)
	Administration	Department of agriculture (national, local)
Old age	Senior organisations	Association of older adults
	Expert organisation	Gerontology association
	Community-based cooperation	Neighborhood network/caring communities
Research & development	Researchers Implementation Scientists	In Switzerland two types: Universities, Universities of Applied Sciences Research institutes (run by government bodies, foundations, etc.)
Politics	Politician	Elected person (parliament, government; municipal, state, and federal level)
Media	Journalist	Professional reporting on care farming development involving older people

When considering this mix of stakeholders, we observe that each brings a distinct perspective on the situation. For example, in agriculture, the production of goods is required and regulated at the national level (e.g., in Switzerland by local or cantonal regulators, based on federal requirements). Additionally, agriculture is increasingly diversified, not only to produce food but also to provide ecosystem services, landscapes (including tourism and recreation), and biodiversity [24]. Thus, when a farm’s production objectives required by regulations are not achieved, care farming – which needs staffing and infrastructure resources – might be seen as an obstacle for the agriculture sector. From a long-term care perspective, however, new care models are required. Yet in this sector, there are other regulatory norms, such as the protection of older people. Here, regulators might view care through the lens of risks (e.g., fall injuries). In contrast, the public health view focuses on prevention and wellbeing outcomes of care farming (e.g., muscle strength through movement). Gerontological associations and senior representatives point out that care farming can support self-determination and quality of life.

Appreciating and analyzing all these diverse perspectives requires a broad view of the topic and an understanding of various interests. For policy purposes, the following graph (see Figure 1) synthesizes these perspectives and highlights areas of action, which are framed by broader concepts in this process. The purple circle marks the overall goal of a policy “care farming with older people”, the left-hand circles (blueish) indicate research tasks, the right-hand (reddish) circles refer to activities on policy level, and the green frame synthesizes core concepts.

**FIGURE 1:**
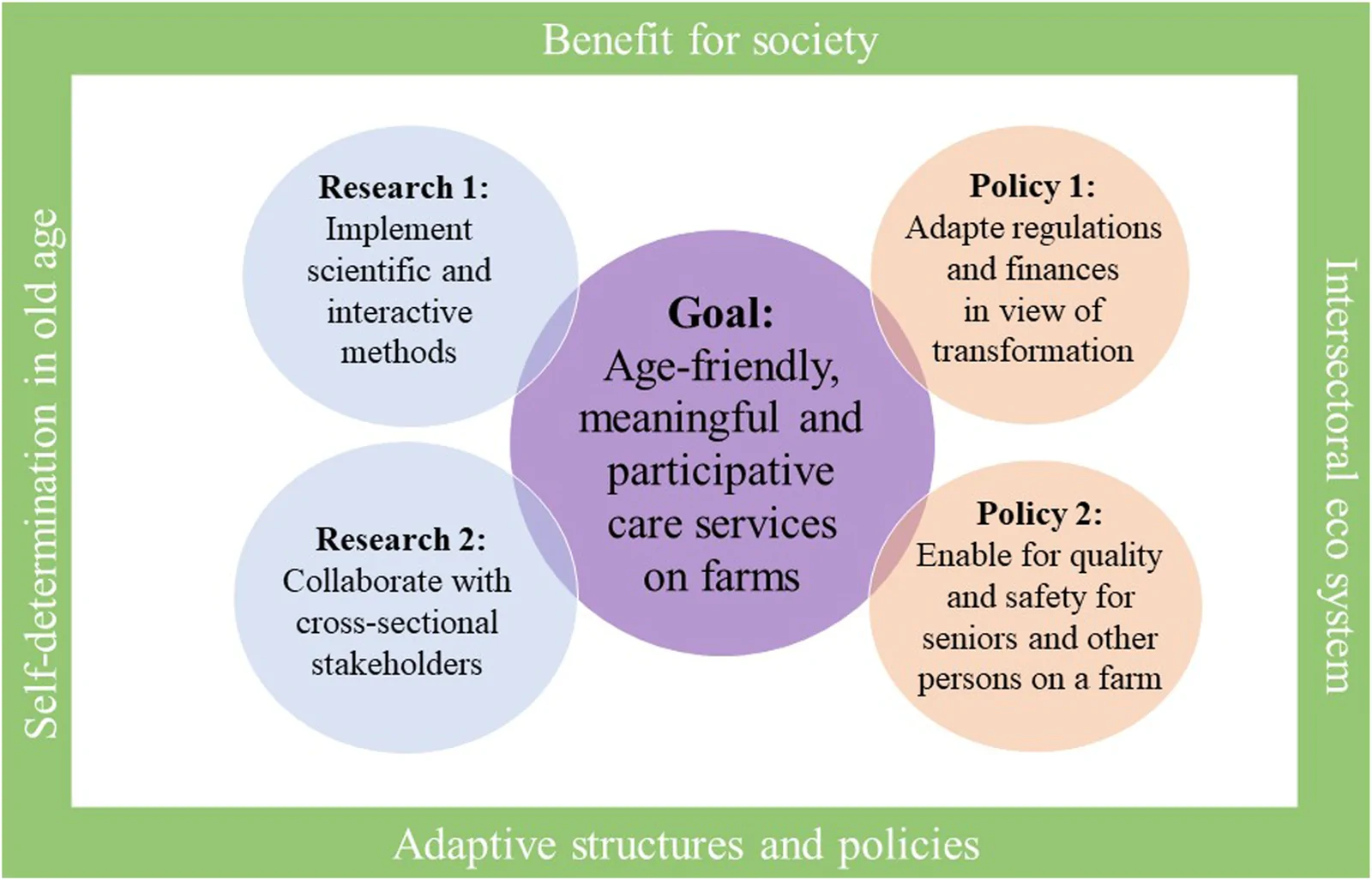
Areas of action framed by core concepts for care arming with older people (own illustration, Switzerland, 2026).

Considering these areas of action, the following points are crucial for policymakers:

- Opinions and behaviors of heterogeneous stakeholders characterize care farming as an intersectoral journey. This can be fruitful and advance the objectives of care farming, yet it may also lead to conflicting and divergent ideas.
- Dialogue is key to bringing together opinions and to negotiating objectives towards sustainable care farming for older adults. Creative formats help express opposing opinions, identify challenges, and develop possible solutions together.
- Preparing for elder-care farming includes getting acquainted with the many regulations in both the agricultural and long-term care sectors, e.g., restrictions on using farmland or buildings and barns for new purposes, or requirements to safeguard people with illnesses such as dementia.
- In this context, the cost-effectiveness of the services must also be considered, possibly with support and guidance for individual farms and care institutions or their respective professional associations.
- To highlight the quality and safety of care farming services and to acknowledge the vulnerability of some elders is essential, since living on a farm is not always idyllic–physically, mentally, or socially. This is particularly important to consider in view of the dark history of abuse and neglect of people of all ages placed involuntarily on farms.

## Conclusion

Learning from stakeholders and accounting for local characteristics provides a basis for the future development of care farming with older people. In this process, sustainable policies should acknowledge the complexity of the intersectoral influences and dynamics [25]. Therefore, for policy development, the agriculture and healthcare sectors should collaborate systematically through a transdisciplinary approach. With this mindset, problems can be framed, and stakeholders are engaged in a meaningful, creative, and significant manner from early adoption until sustainable practice. Case studies and living labs are methods for understanding the characteristics and dynamics of this intersectoral innovation [26]. Simultaneously, robust practices and models of care farming with older persons are essential for a heterogeneous population aged 65 and older. A mindset of experimentation and integration across care farming providers, administrations, associations, and politics can be fruitful for conceptualizing and implementing care farming in a rapidly graying society in Switzerland and elsewhere.
